# Hyper IgE recurrent infection syndrome in South Asia: is there a different outcome?

**DOI:** 10.1186/s13223-018-0292-3

**Published:** 2018-11-02

**Authors:** Rajiva de Silva, Dhanushka Dasanayake, Manouri Senanayake, Ramya Ediriweera, Savithri Dias, Chandima Karunatilleke, Karen Brocas, Fariba Tahami, Suranjith Seneviratne

**Affiliations:** 1 Medical Research Institute, Colombo, Sri Lanka; 2 Lady Ridgeway Hospital, Colombo, Sri Lanka; 3 General Hospital, Kalutara, Sri Lanka; 4 Institute of Infection and Immunity, Royal Free Hospital, London, UK

**Keywords:** Hyper IgE syndrome, Job’s syndrome, Autosomal dominant hyper IgE, STAT3 mutation, Primary immunodeficiency

## Abstract

**Background:**

Hyper IgE recurrent infection syndrome (HIES) is a rare immune deficiency, characterized by recurrent staphylococcal skin and lung abscesses, pneumonia and increased IgE levels. The majority of autosomal dominant HIES (AD HIGE) is due to hypomorphic mutations in the signal transducer and the activator of transcription 3 (STAT3) gene.

**Case presentation:**

Five patients were diagnosed with HIES, based on the clinical criteria and scoring system developed at the National Institutes of Health (NIH), USA. The STAT3 gene was sequenced and previously described mutations were identified in all five patients. We compare the clinical features of our patients with those from Asia, Europe and the US.

**Conclusion:**

Even though the number of patients is limited, there are some clinical differences in patients from South Asia compared to European and even East Asian patients. However, the mutations detected are located at hot spots seen in western and Asian patients with AD HIGE.

## Background

Hyper IgE recurrent infection syndrome (HIES) is a rare immune deficiency, characterized by recurrent staphylococcal skin and lung abscesses, pneumonia and increased IgE levels [1]. The majority of autosomal dominant HIES (AD HIGE) is due to hypomorphic mutations in the signal transducer and the activator of transcription 3 (STAT3) gene [2], resulting in defects in signal transduction for multiple cytokines (IL 6, IL 23) and impaired function of IL 17 [3]. The mutation leads to infectious complications as well as skeletal, dental, facial and connective tissue abnormalities [4]. Defects in tyrosine kinase 2 (TYK2) [5] and homozygous mutations of dedicator of cytokinesis gene (DOCK8) [6] are responsible for autosomal recessive HIGS (AR HIGE). While there are large studies/case series of HIES from the West [1, 7], data from Asia is scarce [8–14] and the prevalence rate unknown. We hypothesized that there may be a different clinical phenotype or evolution in our region, and that we may have different mutations in the STAT3 gene. We report 5 cases of AD HIGE from Sri Lanka that were identified using clinical criteria and the scoring system developed at the National Institutes of Health, USA [15]. All 23 exons and exon/intron boundaries of STAT3 were amplified by PCR and the purified PCR products were sequenced as previously described [16]; all had mutations in the STAT3 gene.

## Case presentation (Tables 1, 2)

### Patient 1

**Fig. 1:**
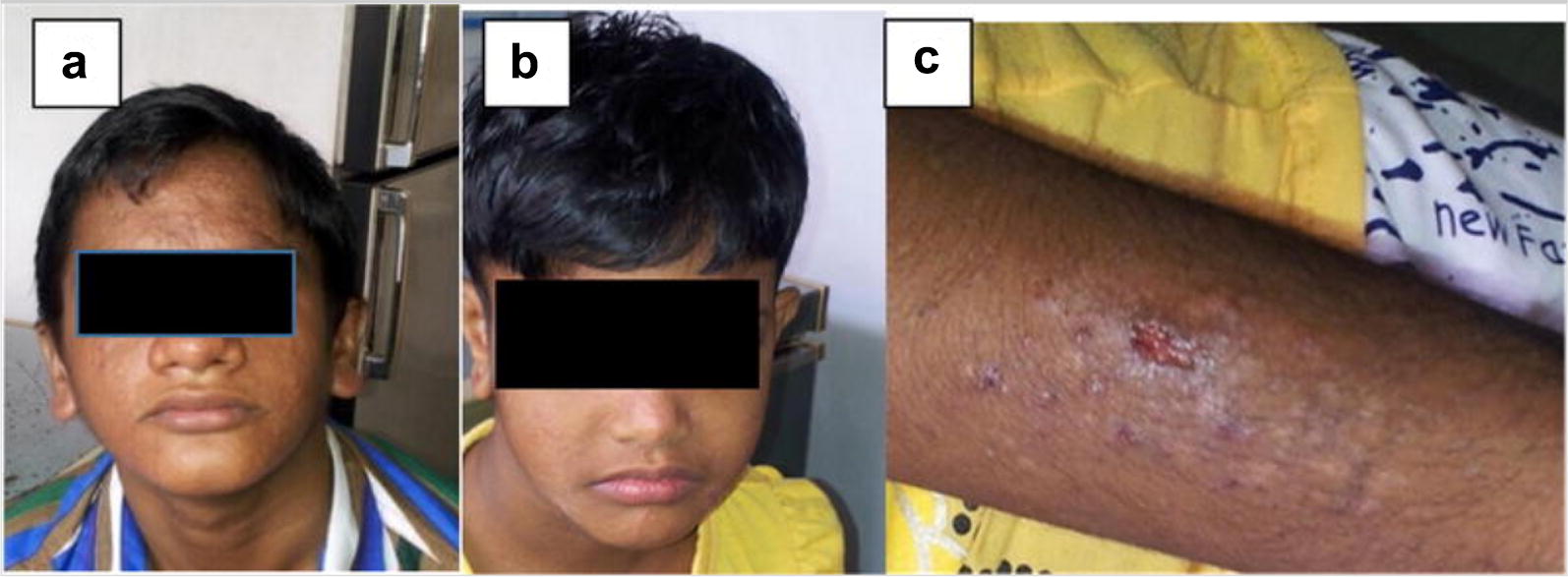
Patients with AD HIGE. **a** Patient 1, **b** patient 2, **c** skin manifestations of patient 2

**Fig. 2:**
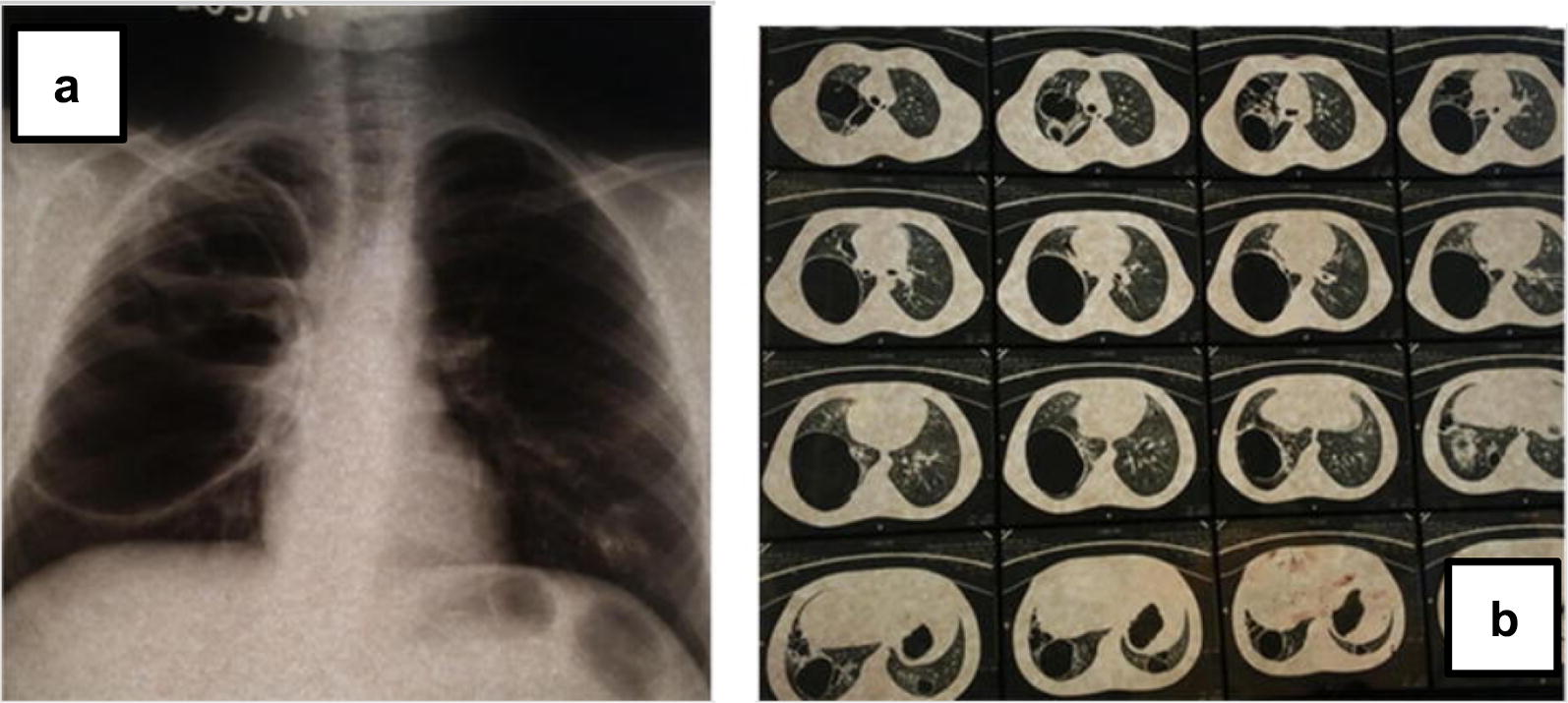
Radiological manifestations of patient 1. **a** Chest X ray and **b** High-resolution computed tomography (HRCT) of chest

A 15 year boy (Fig. 1a) presented with recurrent infections from infancy. He had lower respiratory tract infection (LRTI) with pyopneumothorax at the age of 3 months. He had recurrent (LRTI), scalp and oral abscesses, bilateral otitis media and oral thrush. He developed a generalized rash in the neonatal period and seborrhoeic dermatitis at the age of 5 months. He suffered fractures of the radius and ulna of both arms with minor trauma at 7, 10 and 13 years. He retained one primary tooth. X ray and CT scans of the chest at 15 years revealed a bulky lesion in the right lung (Fig. 2a, b). His serum IgE was elevated (> 2000 IU/ml). His serum IgA was reduced (40 mg/dl, normal range = 70–229). His eosinophil count (250/µl = normal 450), serum IgG, and IgM, isohemagglutinin titre, anti typhoid Vi antibody titre, lymphocyte subsets and nitro blue tetrazolium (NBT) assay were normal. The National Institutes of Health (NIH) score was 70 (Table 3).

**Table 1:** Clinical features

	Patient 1	Patient 2	Patient 3	Patient 4	Patient 5
Age at diagnosis and sex	15 years, male	5 years, female	9 years, female	7 years, female	11 years, male
Age at onset	Neonatal period	2 months	Neonatal period	Neonatal period	4 months
Neonatal skin rash	Yes	No	Yes	Yes	No
Dermatitis	Yes	Yes	Yes	Yes	Yes
Candidiasis	Oral	Oral	No	No	No
Respiratory infections	Recurrent LRTI, pyo-pneumothorax at 3 months Recurrent OM	Recurrent URTI, OM	Recurrent OM	Recurrent OM Recurrent pneumonia (×3), post varicella pneumonia	Pyo-pneumothorax
Pneumatoceles	Yes	No	Yes	Multiple	No
Abscesses	Skin	No	Skin	Skin, peri-pancreatic	Skin, dento-alveolar, lung
Meningitis	No	Yes, recurrent (11 months, 2 years)	No	No	No
Other infections	No	No	No	Pericardial effusion	No
Retained teeth	Yes	No	Yes	Yes	
Fractures	Bilateral radius and ulna	No	No	No	Right ulna

*LRTI* lower respiratory infections, *URTI* upper respiratory infections, *OM* otitis media

**Table 2:** Laboratory Investigations

	Patient 1	Patient 2	Patient 3	Patient 4	Patient 5
Eosinophil count (normal < 450/µl)	250	1000	500	8650	0
Serum IgE level IU/ml	> 2000 (normal 2–629)	> 2000 (normal 2–307)	> 2000 (normal 2–696)	> 2000 (normal 2–403)	> 2000 (normal 2–696)
Serum immunoglobulins (mg/dl)					
IgG (range)	>1120 (726–1085)	1120 (569–1597)	1098 (559–1492)	1212 (559–1492)	985 (779–1456)
IgA (range)	40 (70–229)	57 (55–152)	153 (54–221)	161 (54–221)	44 (2–208)
IgM (range)	> 94.8 (35–72)	> 94.8 (22–100)	264 (27–118)	42 (27–118)	>94.8 (35–132)
Isohemagglutinin titre (normal > 1: 10 dilution)	Normal	Normal	Normal	Normal	Normal
Typhoid Vi IgG EU/Ml (Sero-positive ≥ 150 EU/ml)	> 600	Not done	503	Not done	312
Lymphocyte subsets (/µl)					
CD 3 (normal range)				12,920 (2300–6500)	
CD 4 (normal range)	42% = 1290 (400–2100)	32% = 1760 (300–2000)	34% = 1478 (9300–2000)	4718 (1500–5000)	36% = 971 (400–2100)
CD 8 (normal range)	35% = 1075 (200–1200)	36% = 1980 (300–1800)	31% = 1341 (399–1200)	7461 (500–1600)	27% = 749 (200–1200)
CD 19 (normal range)	13% 399 (200–600)	20% = 1100 (200–1600)	23% = 995 (200–1600)	4125 (600–3000)	13% = 361 (200–600)
Mutation analysis of STAT3 gene	A heterozygous mutation in 1145 G to A in exon 13 leading to an amino acid change R382Q in the DNA binding domain	A heterozygous mutation in 1145 G to A in exon 13 leading to an amino acid change R382Q in the DNA binding domain	A heterozygous mutation in 1909 G to A in exon 21 leading to an amino acid change V637M in the SH2 domain	A heterozygous mutation in 1144 C to T in exon 13 leading to an amino acid change R382W in the DNA binding domain	A heterozygous mutation in 1909 G to A in exon 21 leading to an amino acid change V637M in the SH2 domain

**Table 3:** NIH Score based on [14]

Feature	Score				
	Case 1	Case 2	Case 3	Case 4	Case 5
Highest serum IgE level	10	10	10	10	10
Skin abscess	8	0	8	8	8
Pneumonia	8	0	0	6	0
Parenchymal lung abnormalities	8	0	8	8	6
Retained primary teeth	1	0	2	2	0
Scoliosis	0	0	0	0	0
Fractures with minor trauma	8	0	0	0	4
Highest eosinophil count	6	6	0	6	0
Characteristic face	5	2	0	5	0
Midline anomaly	0	0	0	0	0
Newborn rash	4	0	4	4	0
Eczema	4	2	1	0	0
URTI/year	4	4	0	0	0
Candidiasis	1	1	0	0	0
Other serious infections	0	4	0	4	0
Fatal infections	0	0	0	4	0
Hyperextensibility	0	0	4	0	0
Lymphoma	0	0	0	0	0
Increased nasal width	1	0	0	0	0
High palate	2	0	0	2	0
Young age correction					
Total	70	29	37	59	28
STAT3 mutation	Yes	Yes	Yes	Yes	Yes

A heterozygous mutation with a nucleotide exchange of 1145 G to A in exon 13 leading to an amino acid change R382Q in the DNA binding domain identified in the STAT3 gene.

### Patient 2

A 5 years old girl (Fig. 1b, c) (sister of case 1) presented with recurrent infections, including 2 episodes of meningitis at 11 months and 2 years, several episodes of eczema herpeticum and oral thrush. She also had recurrent episodes of upper respiratory tract infections and otitis media. She developed a generalized skin rash at 2 months of age. Her serum IgE was elevated (> 2000 IU/ml) and she had eosinophilia (1000/µl, normal = 450/µl). Her serum immunoglobulins, isohemagglutinin titre, lymphocyte subsets and NBT assay were normal. The anti typhoid Vi vaccine was not done. The National Institutes of Health (NIH) score was 29 (Table 3).

A heterozygous mutation with a nucleotide exchange of 1145 G to A in exon 13 leading to an amino acid change R382Q in the DNA binding domain identified in the STAT3 gene.

### Patient 3

A 9 year old girl presented with recurrent infections from infancy. This included umbilical sepsis and a generalized skin rash during the neonatal period, recurrent skin abscesses since 6 months of age and recurrent episodes of otitis media. She has retained 2 primary teeth. Chest X ray revealed of a right upper lobe pneumatocele. She was diagnosed with the β thalassaemia trait. Her serum IgE was elevated (> 2000 IU/ml), while her eosinophil count was mildly increased (500/µl, normal = 450/µl). Her serum immunoglobulins, isohemagglutinin titre, anti typhoid Vi antibody titre, lymphocyte subsets and NBT assay were normal. The National Institutes of Health (NIH) score was 37 (Table 3).

A heterozygous mutation with a nucleotide exchange of 1909 G to A in exon 21 leading to an amino acid change V637M in the SH2 domain identified in the STAT3 gene.

### Patient 4

A 7 year old girl presented with recurrent infections from the neonatal period, including septicemia on day 9 after birth with *S. aureus* isolated from blood, recurrent skin abscesses which were drained on 5 occasions, 3 episodes of pneumonia, several episodes of otitis media and chickenpox which was complicated by post varicella pneumonia at 7 years of age. She also developed a rash on the scalp and face in the neonatal period. Her chest X ray showed multiple pneumatoceles. She has had a pericardial effusion, and a peripancreatic abscess diagnosed by ultra sound. Both were aspirated. She had retained 2 primary teeth. She died at 7 years 5 months following a brief respiratory tract infection. Her serum IgE was elevated (> 2000 IU/ml), while her eosinophil count was increased (8650/µl), normal = 450/µl. Her serum immunoglobulins, isohemagglutinin titre, lymphocyte subsets and NBT assay were normal. The anti-typhoid Vi vaccine was not performed as the patient died before the post vaccine sample was collected. The National Institutes of Health (NIH) score was 59 (Table 3).

A heterozygous mutation with a nucleotide exchange of 1144 C to T in exon 13 leading to an amino acid change R382W in the DNA binding domain was identified in the STAT3 gene.

### Patient 5

An 11 year old boy presented with recurrent abscesses since 4 months of age, including skin, dento-alveolar and lung abscesses. He developed a pyo-pneumothorax at 3½ years, and the aspirated pus grew *P. aeruginosa*. He had also developed a skin rash at the age of 6 months. He had a greenstick fracture of the right ulna at 6 years. His serum IgE was elevated (> 2000 IU/ml). His eosinophil count, serum immunoglobulins, isohemagglutinin titre, anti typhoid Vi antibody titre, lymphocyte subsets and NBT assay were normal. The National Institutes of Health (NIH) score was 28 (Table 3).

A heterozygous mutation with a nucleotide exchange of 1909 G to A in exon 21 leading to an amino acid change V637M in the SH2 domain identified in the STAT3 gene.

The laboratory investigations and STAT3 mutations detected in our patients are presented in Table 2. STAT3 mutations are presented in figure. Table 4 shows a comparison of our patients, with those from the west [1, 7] India [8], ethnic Chinese from China [9, 10], Hong Kong [11] and Taiwan [12], and Japanese patients [13].

**Table 4:** Comparison of US, European and Asian patients with STAT3 mutations positive AD-HIGE

Clinical feature	US (n = 64) [16]	French (n = 60) [7]	Indian (n = 04) [8]	Ethnic Chinese (n = 12) [9–13]	Japanese (n = 08) [14]	Sri Lankan (n = 05)
Age range		1 month–46 years	4 months–35 years	1.7–26 years	6–21 years	5–15 years
	%	%	%	% (n)	% (n)	%
Findings related to infections and immune system						
Moderate to severe eczema	90.6	nm	nm	91.6 (11/12)	87.5 (7/8)	60
Serum IgE > 2000 IU/ml	nm	96	100	75 (9/12)	100 (8/8)	100
Eosinophilia (> 2 SD above normal mean)	70.7	80	75	91.6 (11/12)	75 (6/8)	60
Recurrent pneumonia, X-ray proven	95	nm	50	75 (9/12)	87.5 (7/8)	40
Recurrent skin abscesses	90.6	nm	50	100 (12/12)	75 (6/8, 2 = 1–2 abscesses)	80
Recurrent (> 3/year) upper respiratory infections	45.8	nm	nm	71.4 (7/14)	37.5 (3/8)	80
Mucocutaneous candidiasis	43.1	85	nm	58.3 (7/12)	67.5 (5/8)	40
Pneumatoceles	74.6	52	25	66.6 (8/12)	67.5 (5/8)	60
Newborn rash	64.9	48	50	66.6 (8/12)	100 (8/8)	60
Other serious infections	nm	43	0	66.6 (8/12)	25 (2/8)	40
Lymphoma, other cancer	03	7	0	0	0	0
Findings related to bones, teeth, and connective tissue						
Characteristic face	90.6	95	75	91.6 (11/12)	100 (8/8)	60
Retained primary teeth	80	65	nm	100 (5/5)	37.5 (3/8)	60
Hyperextensibility of joints	52.7	50	nm	58.3 (7/12)	50 (4/8)	20
Increased nasal width (interalar distance > 1 SD above normal value for age, race)	51.9	85	nm	80 (4/5)	12.5 (1/8)	20
Scoliosis > 10°	26	38	nm	0	25 (2/8)	60
Recurrent fractures following minor trauma	45.8	42	nm	8.3 (1/12)	25 (2/8)	40
High-arched palate	54.7	53	nm	33.3 (3/12)	25 (2/8)	40
Congenital skeletal anomalies	nm 10	nm	nm	0	nm	0
Focal hyperintensities on brain MRI	nm	< 1	nm	nm	nm	ne
Chiari type I malformation	nm	nm	nm	nm	nm	ne
Coronary vasculature abnormalities	nm	nm	nm	nm	nm	ne

*nm* not mentioned, *ne* not evaluated

## Discussion

All our patients were from the majority Sinhalese community (who form 74% of the country’s population). A previous case report of AD HIGE from Sri Lanka was also from this community [17]. Eighty percent of our patients had recurrent skin abscesses, and this was similar to the European [7, 16], Chinese [9–13] and Japanese cohorts [14]. On the other hand, only 50% of four reported Indian patients with AD HIGE had recurrent abscesses [8]. One of our patients developed a peripancreatic abscess, while another developed a lung abscess, and both were successfully drained. We could not find other reports of a peripancreatic abscess in AD HIGE. Mucocutaneous candidiasis was seen in 40% of our patients, compared to ≥ 70% of French [7] and > 50% of Chinese [9–13] and Japanese patients [14]. Patients from the US with STAT3 mutations had figures similar to ours [15]. Recurrent pneumonia was seen in ≤ 50% of Sri Lankan and Indian [8] patients, whereas these were noted in > 75% of ethnic Chinese [9–13], and Japanese patients [14] and in the US [16]. One of our patients (case 4) developed pneumonia following varicella. Severe viral infections are typically seen in the AR HIGE [18], and chicken pox pneumonia has rarely been reported in AD-HIGE [7]. One of our patients (case 2) had two episodes of bacterial meningitis. Although meningitis has been reported in AD HIGE [7, 19] recurrent episodes are very rare, with one patient previously reported from India [20]. However, mutation analysis was not performed in that patient. Interestingly, our patient also had several episodes of eczema herpeticum, which is usually seen in patients with the AR HIGE [18]. Vascular malformations and neurological abnormalities are common in AD HIGE patients from the west [21], but were not seen in our patients and in other Asian cohorts [8–14]; however the lack of active detection may be a possible reason for this. High IgE levels are seen in ≥ 95% of European [7] and Asian patients [8, 14]. Eosinophilia was seen in 60% of our patients; the figures have been higher in other cohorts. Eosinophil counts may fluctuate in time, and the lower value in our cohort may not be a true representation. One patient had partial IgA deficiency, but seroconverted following typhoid Vi vaccination, and had isohemagglutinins. IgA and IgG deficiency has been rarely reported in AD HIGE [22]. Impaired specific antibody responses may occur in some AD HIGE patients [23]. All our patients had isohemagglutinins and all three patients immunized with the typhoid Vi polysaccharide vaccine made good antibody responses.

The mortality rate seems to be higher in South Asia. The son of one of the patients with AD HIGE from India who had typical features but was not included in the study [8], as well as one of our patients expired following infections, a mortality rate of 20% in both instances. The mortality rate was 5% in the French study (another patient had died following an accident) [7]. None of the ethnic Chinese or Japanese patients with STAT3 mutations had died [9–14]. However, 3 of 4 patients with phenotypic AD-HIGE with wild type STAT3 from Taiwan died following a myocardial infarction due to coronary aneurisms, complications from a lymphoma and sepsis [12]. More advanced health care facilities in South East Asia and Europe may be responsible for the better outcomes in these regions.

**Fig. 3:**
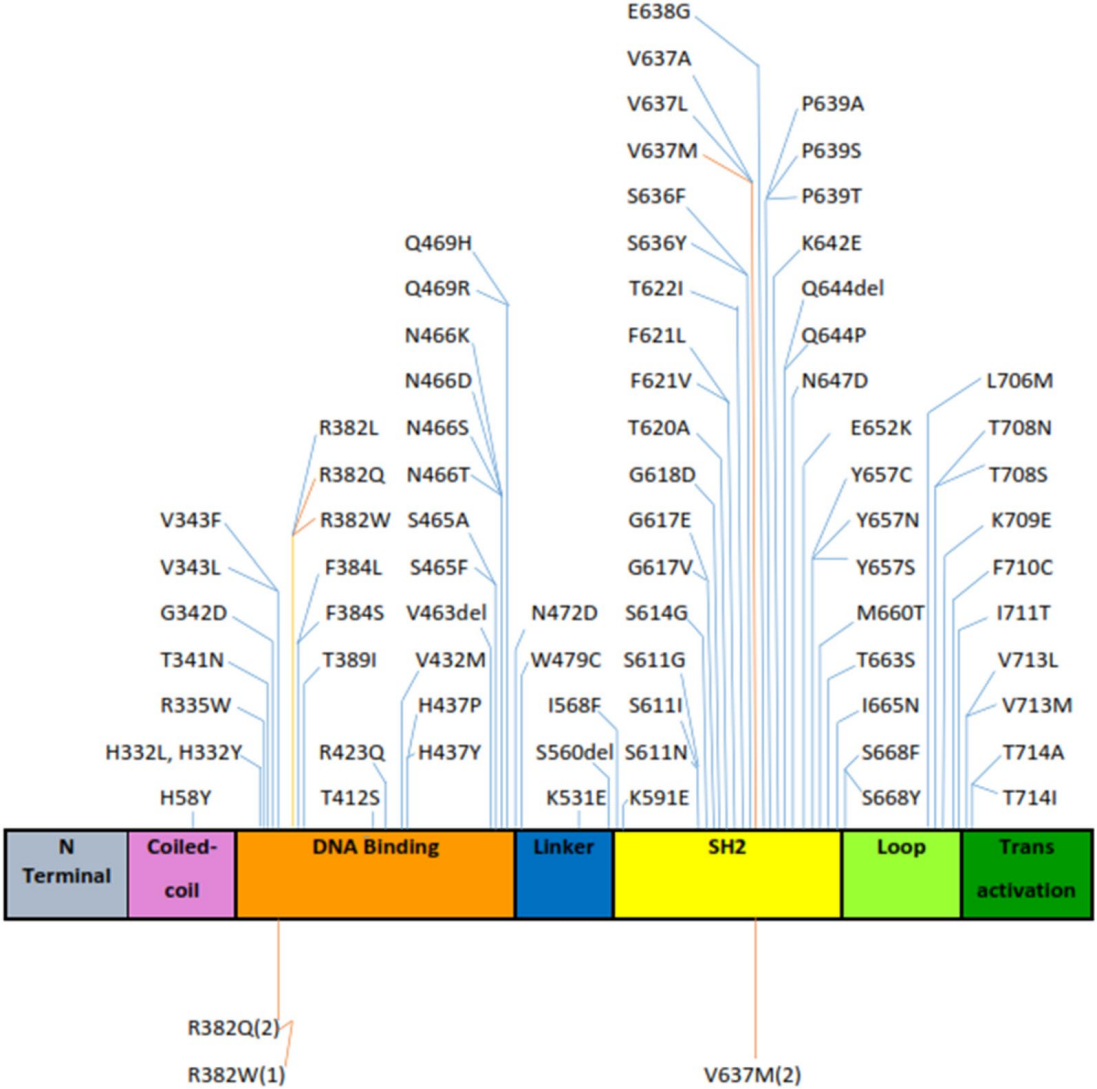
Schematic structure of STAT3. Previously described mutations [7] are shown in the upper part of the figure. Patients identified in this study with the STAT3 mutation (and number) are shown in the lower part of the figure

The mutations in our patients have been recognized previously (Fig. 3, [7]) and were found to be involved with the DNA binding and SH2 domains of the STAT3 gene. There are several hot spots identified in previous studies, including Arginine (R) at position 382 of the DNA binding domain and Valine (V) at position 637 in the SH2 domain [2]. Three of our patients had mutations at position 637 and the other two at position 382. The 1144 C-T, R382W transition has been identified in black, Hispanic and white individuals in the US [2] and in Japanese patients [14]. It was seen in almost half the patients in one study [2]. The 1145 G–A, R382Q transition has been identified in Indian [8], Chinese [9], Japanese [14], black and white patients in the US [2] and the 1909 G–A, V637M transition in Chinese [9] as well as white individuals [2]. The DNA binding region hot spot at arginine 382 may be associated with a higher mortality, as 5 of 7 patients who died due to infection related causes in one study had this mutation, even though it was not statistically significant [24]. The patient who died in our study (no: 4) carried the same mutation. Further studies may be needed in this regard.

## Conclusion

The mortality rate seems to be higher in South Asia compared to East Asia and the West and recurrent pneumonia is less common in the sub-continent. However, the mutations detected are located at the same hot spots seen in Western and East Asian AD HIGE patients. Less advanced health care facilities in South Asia may be responsible for the worse outcomes in this regions.
